# A novel signature based on immune‐related gene pairs and clinical features to predict prognosis and treatment effect in “driver gene negative” lung adenocarcinoma

**DOI:** 10.1002/cam4.4577

**Published:** 2022-03-04

**Authors:** He‐yuan Cai, Hao‐shuai Yang, Shi‐chao Shan, Yi‐yan Lei, Jian‐yong Zou, Ying Zhu, Hong‐he Luo

**Affiliations:** ^1^ Department of Thoracic Surgery The First Affiliated Hospital, Sun Yat‐sen University Guangzhou China; ^2^ Department of Radiology The First Affiliated Hospital of Sun Yat‐sen University Guangzhou China

**Keywords:** “driver gene negative” lung adenocarcinoma, immune‐related gene, immunotherapy, nomogram

## Abstract

**Objective:**

Examining the role of immune‐related genes (IRGs) in “driver gene negative” lung adenocarcinoma (LUAD) may provide new ideas for the treatment and study for this LUAD subgroup. We aimed to find the hub immune‐related gene pairs can stratify the risk of “driver‐gene‐negative” LUAD.

**Materials and Methods:**

IRGs were identified according to ImmPort database based on RNA sequencing results of tumors and normal tissues from 46 patients with “driver gene negative” LUAD at The First Affiliated Hospital of Sun Yat‐sen University and cyclically singly paired as immune‐related gene pairs (IRGPs). Multivariate Cox analysis was used to construct an immune risk model and a prognostic nomogram combining was also been developed. Immune microenvironment landscape described by CIBERSORT and drug sensitivity calculated by pRRophetic algorithm were used to explore possible treatment improvements.

**Results:**

A novel immune risk model with 5‐IRGPs (CD1A|CXCL135, CD1A|S100A7L2, IFNA7|CMTM2, IFNA7|CSF3, CAMP|TFR2) can accurately distinguish patients in the high‐ and low‐risk groups. Risk score act as an independent prognostic factor and is related to clinical stage. There are significant differences in tumor immune microenvironment and PD‐1/PD‐L1/CTLA‐4 expression between groups. The low‐risk patient may benefit more from the commonly used chemotherapy regimens such as gemcitabine and paclitaxel.

**Conclusion:**

This study constructed 5‐IRGPs as a reliable prognostic tool and may represent genes pairs that are potential rationale for choice of treatment for “driver gene negative” LUAD.

## Graphical abstract

There are few studies on “Driver‐gene‐negative” lung adenocarcinoma. It’s the first time that we did RNA‐seq on these patient samples to explore immune characteristics and construct a prognostic prediction model.

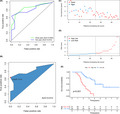

## 1 INTRODUCTION

Lung adenocarcinoma (LUAD) is the most common pathological type of lung cancer, accounting for more than 40% of the total number of lung cancer cases.^1^, ^2^ Over the last decade, with the continuous exploration of tumor driver genes, ALK, EGFR, HER2, KRAS, MET, BRAF, ROS1, and RET have been found as driver genes in the field of lung adenocarcinoma.^3^ Targeted therapeutic drugs based on driver genes have been developed in decades, which has resulted in a survival benefit for patients with “driver gene positive” LUAD.^4^ However, 10% to 55% of lung adenocarcinomas fail to detect the above known driver gene mutations, we refer to these patients as patients with “driver gene negative” lung adenocarcinoma.^5^, ^6^ Although the Immune checkpoint inhibitors (ICIs) is of great help in the treatment of these patients,^7^ there are still problems such as nonresponse and immune‐related toxicity in the clinical application of existing mainstream immunotherapeutic drugs.^8^

Immune‐related genes (IRGs) play an important role in the development of tumors,^9^ and drugs targeting immune‐related genes are also widely used in clinical practice. A series of immune‐related genes with great clinical potential have been found such as PD‐1/PD‐L1/CTLA‐4,^10^, ^11^ making immunotherapy of tumors an important means. At present, there have been some bioinformatics studies focusing on the mechanism of immune gene action in lung adenocarcinoma,^12^, ^13^ and these studies provide an important reference for immunotherapy of lung adenocarcinoma. However, there is a lack of targeted studies on the immune mechanism of action in “driver gene negative” patients, which are most in need of immunotherapy. The search for new immunotherapeutic modalities and elaboration of the immune mechanisms driving “driver gene‐negative” patients can provide prognostic value.

This study was designed to construct an immune‐related prognostic classifier based on a genome‐wide transcriptome profiling in 46 “driver gene negative” patients from The First Affiliated Hospital of Sun Yat‐sen University, and assessed this classifier from survival rate, clinical features, immune cells infiltrating, immunosuppressed biomarkers, and chemotherapeutic effects, providing new ideas for the treatment of “driver gene negative” patients.

## 2 METHODS

### 2.1 Detection of driver genes and identification of patients

This study included patients with “driver gene negative” LUAD at The First Affiliated Hospital of Sun Yat‐sen University between September 2003 and June 2015. Immunohistochemical staining and immunoblotting assays in the formalin‐fixed, paraffin‐embedded (FFPE) tissues were applied to determine the patient's “driver gene negative” status. The specific steps are as follows:

First, we examined tissue samples from 626 patients with pathologically confirmed LUAD using a 13‐genes panel (including EGFR, KRAS, BRAF, PIK3CA, NRAS, HER2, MET, AKT1, c‐KIT, PDGFRA, ALK, RET, and ROS1) to determine the expression of their driver genes. Then, EGFR and KRAS were validated in those patients using ARMSPCR and ALK was validated using FISH assay. Finally, eventually, we identified 189 patients with LUAD as “driver gene negative” with ALK, EGFR, HER2, KRAS, MET, BRAF, ROS1, and RET mutation negative, which could not utilize current mainstream targeted therapy.^5^

This project was approved by the Ethics Committee and Institutional Review Board of Sun Yat‐sen University and informed consent was waived. A biopsy and immunohistochemistry analysis on the original tumor tissue were performed to confirm the pathological features. We also collected clinical information, such as gender, age, smoking history, TNM stage, and differentiation of the tumor. The AJCC (American Joint Committee on Cancer) 7th edition cancer staging system was used to stage tumors. OS was measured from the date of surgery to the date of death or the last date of follow‐up. Unless the patients passed away, our follow‐up lasted at least 5 months.

### 2.2 Acquisition of genetic data

A total of 60 paired LUAD and precancerous tissue samples were selected randomly from 189 patients with “driver gene negative” LUAD including 15 pairs each in stage I, stage II, stage III, and stage IV. However, 14 pairs were excluded, of which eight pairs were due to degradation of RNA during storage and six pairs were due to missing partial clinical information.

We harvested total RNA from fresh tissue, and analyzed RNA with a Nanodrop2000 spectrophotometer (Thermo Fisher Scientific) and assessed RNA integrity with an Agilent 2100 Bioanalyzer system (Agilent Technologies). According to the Agilent One‐Color Microarray Gene Expression Analysis Protocol (Agilent Technology), we performed sample labeling and array hybridization. Then, 100 μl of hybridization solution was dispensed into spacer slides and assembled into gene expression microarray slides. The 4 × 44 K whole human genome expression microarray (Agilent design ID 026652, GEO accession number GPL13497) was used to get profiles the expression of 27,958 genes in remaining 46 pairs. GeneSpring GX v12.1 software package was used to do quantile normalization and subsequent data processing, and the genes with low expression were excluded for analyses.

### 2.3 Construction of immune‐related genes (IRGPs)

ImmPort database (http://www.immport.org) was used to recognize IRGs. We identified a total of 2498 immune‐related genes in the gene list, of which 1299 genes included in the expression microarray were used for subsequent analysis. The differential expression IRGs (DEIRGs) between tumor and precancerous tissue were identified with the thresholds as false discovery rate (FDR) < 0.05, along with |log2 FC (fold‐change)| > 1.

The DEIRGs were cyclically paired singly, and an IRGP was calculated as follows:

$${\text{IRGP}}_{\mathrm{ab}}=\left\{\begin{array}{cc} 1, & {\mathrm{IRG}}_{a}<{\mathrm{IRG}}_{b}\\ 0 & {\mathrm{IRG}}_{a}\geq {\mathrm{IRG}}_{b} \end{array}\right.$$

If an IRGP_*a*_ unique value of 0 or 1 among all samples, it will be removed to avoid biases and unrepeatability of the study.

### 2.4 Establishment and validation of the risk model

We first initially screened IRGPs using univariate Cox regression, and followed by multivariate Cox regression analysis of selected IRGPs to determine the gene pairs that were finally used to construct the model with a threshold of *p* < 0.05. The AUC values of each curve were calculated and labeled in the Figure. The 3‐ and 5‐year ROC curves of the model were shown in the result. The formula we used to calculate the Risk Score is as followed:

$$\text{Risk Score}={\hat{h}}_{0}\left(t\right)\sum_{i=1}^{k}\text{βiSi},$$

The AIC values of each points of the 5‐year ROC curve were evaluated to identify the maximum inflection point, which was considered as the cut‐off point to distinguish high‐risk and low‐risk groups.

To validate this cut‐off value, we plotted the score distribution dot plot and the survival status dot plot. At the same time, we used Kaplan–Meier survival analysis curves to present survival differences between groups. The process was visualized using the R packages “survival,” “glmnet,” “pHeatmap,” “survminer,” “survivalROC,” and “pbapply.”

### 2.5 Independent prognostic and clinical relevance analysis

We performed a correlation analysis to better understand the relationship between clinical features and Risk Score. In addition, we used univariate and multivariate Cox analyses to explore independent prognostic factors of patients with “driver gene negative” LUAD in depth, including age, gender, stage, TNM stage, and Risk Score.

### 2.6 Establishment and verification of prognosis nomogram

We developed a prognosis nomogram to predict the prognosis of patients with “driver gene negative” LUAD according to the results of multivariate Cox regression. The nomogram was built on two predictors, Risk Score and tumor stage. Calibration curves were plotted and the 3‐year‐AUC and 5‐year‐AUC were shown to assess the performance of the nomogram. Kaplan–Meier analyses were performed to assess survival differences between groups, and a log‐rank test for statistical comparison.

### 2.7 Investigation of tumor immune microenvironment

We used CIBERSORT to calculate the immune infiltration statues between two groups and the difference were analyzed by Wilcoxon signed‐rank test. We used Spearman correlation analysis to interrogate the association between Risk Score and immune cell infiltration. At the same time, we also compared the expression of commonly used checkpoint genes, such as PD‐1, PD‐L1, and CTLA‐4 between two risk groups. The procedure was performed using R package “ggplot2”.

### 2.8 Evaluation of the sensitivity of chemotherapeutic agents

In order to explore the difference in sensitivity to chemotherapeutic drugs between high‐risk and low‐risk groups, we applied “pRRophetic” package in R to predict the half‐maximal inhibitory concentration (IC50) of different chemotherapeutic drugs in each patient.^14^ This package could predict IC50 by creating statistical models from the drug sensitivity and RNA‐seq data based on Genomics of Drug Sensitivity in Cancer (GDSC) (www.cancerrxgene.org/).

## 3 RESULTS

### 3.1 Clinical features of 46 patients with “driver gene negative” LUAD

Totally 46 patients' samples with “driver gene negative” LUAD randomly selected according to clinical stage, the overall survival was 3.13 ± 1.62 years, and 13 patients died during the follow‐up time (median: 2.83 years). The incidence of lung adenocarcinoma was similar in males and females, aged from 28 to76 years (median: 57.5 years). More other pathological features are shown in Table 1.

**TABLE 1:** Clinical information of patients

Variables	Number of patients (%)
Status	
Dead	13 (28.26)
Alive	33 (71.74)
Gender	
Male	22 (47.83)
Female	24 (52.17)
Age	
<60	28 (60.87)
≥60	18 (39.13)
Clinical stage	
I	13 (28.26)
II	13 (28.26)
III	11 (23.91)
IV	9 (15.57)
T stage	
T1	21 (45.65)
T2	17 (36.96)
T3	4 (8.70)
T4	4 (8.70)
N stage	
N0	19 (41.30)
N1	9 (15.57)
N2	12 (26.09)
N3	6 (13.04)
M stage	
M0	38 (82.61)
M1	8 (17.39)
Overall survival	
Years (mean ± SD)	3.13 ± 1.62

### 3.2 Identification of DEIRGs

**FIGURE 1:**
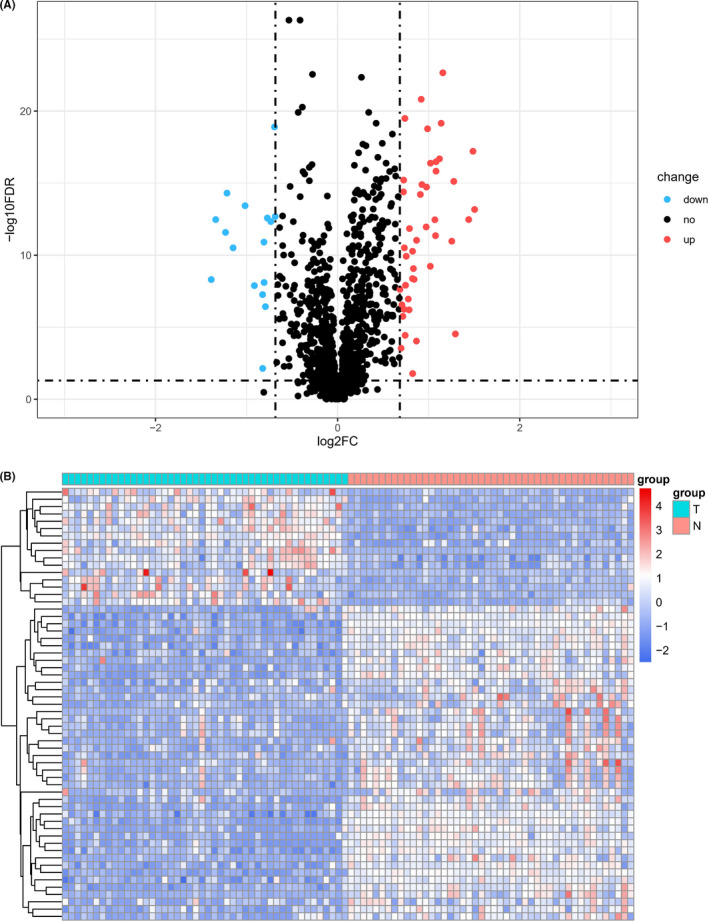
Volcano plot and heatmap of differentially expressed immune‐related genes in 46 patients of “driver gene negative” LUAD

Immune‐related genes (IRGs) of 1299 were recognized in expression microarray from 46 patients with “driver gene negative” LUAD. And 59 genes were identified as differentially expressed immune‐related genes (DEIRGs) which expressed differently between normal tissue and tumor tissue. Of the 59 differentially expressed genes, 43 were highly expressed, and 16 were lowly expressed. The volcano plot and heat plot of DEIRGs are shown in Figure 1.

### 3.3 A 5‐IRGPs signature can predict the prognosis of patients with “driver gene negative” LUAD

Through iterative cycle with 0‐or‐1 gene screening, 1175 IRGPs were screened from 59 DEIRGs. Combined with clinical survival data, the univariate Cox regression analysis showed that 9‐IRGPs were prognostic risk factors of LUAD patients (*p* < 0.05). Further multivariate Cox regression analysis suggested 5‐IRGPs, which are CD1A|CXCL135, CD1A|S100A7L2, IFNA7|CMTM2, IFNA7|CSF3, CAMP|TFR2 could predict the prognosis of patients with “driver gene negative” LUAD (*p* < 0.05), and all of the 5‐IRGPs were risk factors of prognosis with hazard ratio less than 1 (Supplementary Table S1).

**FIGURE 2:**
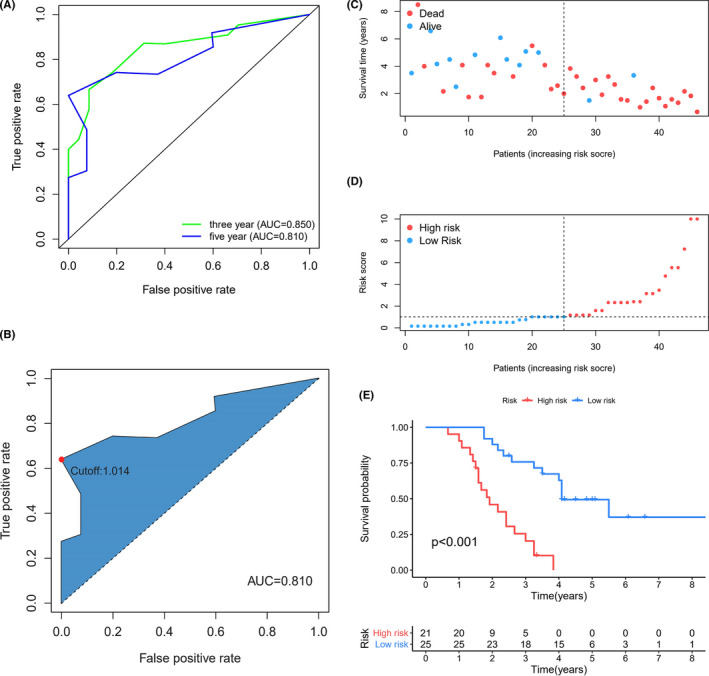
Construction of prognostic model with five DEirRNA pairs signature of 46 patients of “driver gene negative” LUAD. (A, B) Time‐dependent ROC curves of prognostic model and maximum inflection point. (C, D) Distribution of risk score and survival status of the five prognostic DEirRNA pairs. (E) Kaplan–Meier curves of OS in low‐risk and high‐risk group

By using the formula in the method, we calculated the Risk Scores of 46 patients to further evaluate the predictive performance of immune signature. Time‐dependent ROC survival curve showed that the signature had a good prediction performance with 0.850 of 3‐year‐AUC and 0.810 of 5‐year‐AUC (Figure 2A). Meanwhile, the maximum inflection point of 5‐year ROC curve was taken as the cut‐off point (cut‐off:1.014) (Figure 2B), then we divided 46 patients into high‐risk group (*n* = 21) and low‐risk group (*n* = 25) by the cut‐off. The Risk Score distribution point plot and survival status point plot showed that the survival time and survival status of low‐risk group were better than those of high‐risk group (Figure 2C,D). Furthermore, Kaplan–Meier survival curves showed that the 3‐years and 5‐years survival rates were both higher in low‐risk group than high‐risk group (*p* < 0.001) (Figure 2E), suggesting a better prognosis in the low‐risk group.

### 3.4 Risk score is clinically relevant and act as an independent prognostic factor

We also explored the relationship between Risk Score of the signature and clinical characteristics and prognosis for 46 patients. Risk Score, age, gender, tumor stage, and TNM stage were all included in Cox regression analysis. Univariate Cox regression analysis showed that Risk Score, tumor stage, T stage, M stage, and N stage were associated with prognosis of LUAD patients (*p* < 0.05), and multivariate Cox regression analysis only showed that Risk Score was an independent prognostic risk factor in patients with “driver gene negative” LUAD (*p* < 0.05) (Table S2).

**FIGURE 3:**
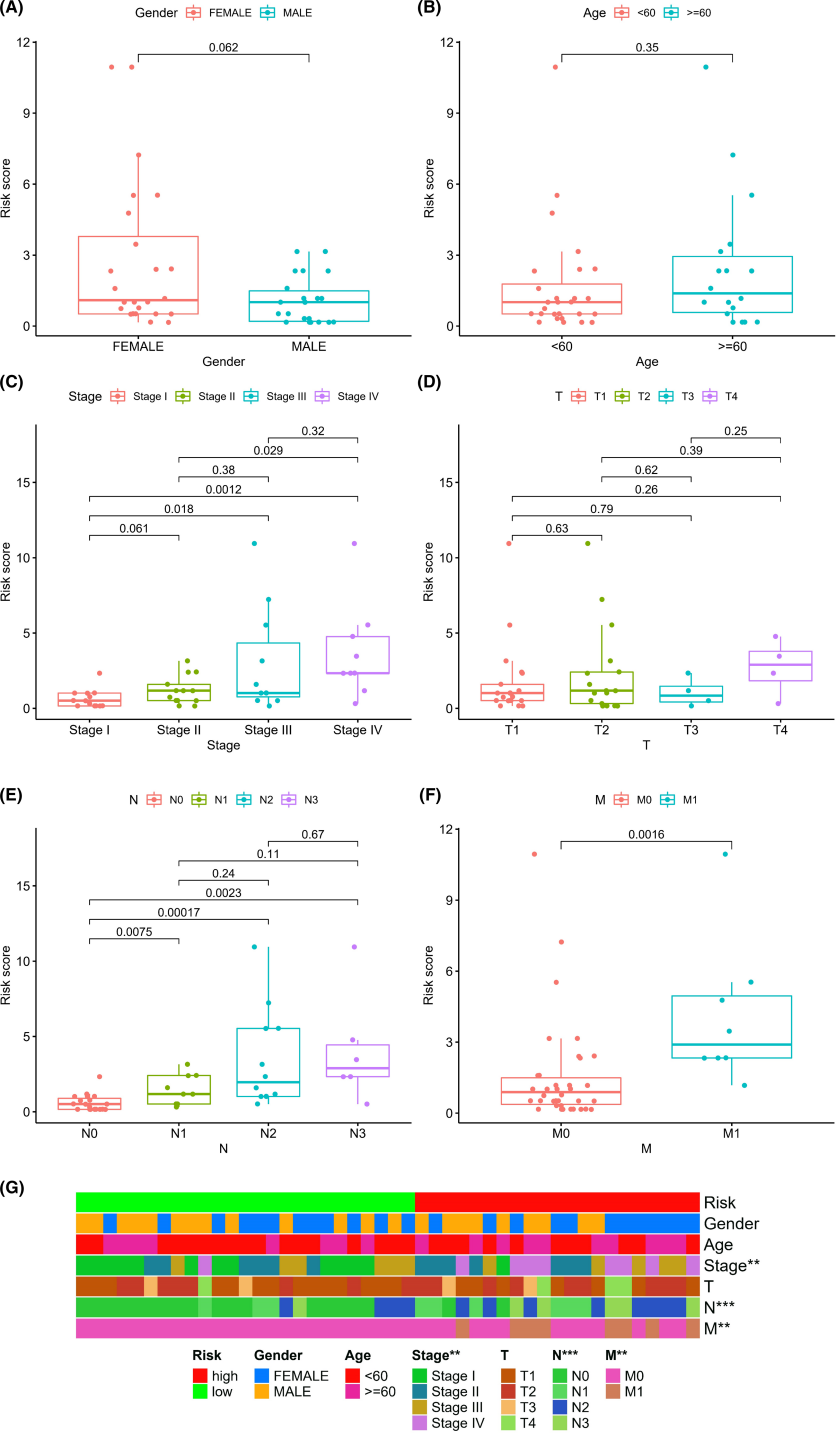
The predictive performance of the signature and clinical characteristics and correlations between risk score and clinical characteristics. (A, B) ROC curve of 3 and 5 year in signature and clinical characteristics. (C–H) Correlations between risk score and clinical characteristics. (I) Heatmap of low‐ and high‐risk group and clinical characteristics. *p < 0.05, **p < 0.01, and ***p < 0.001

We also explored the correlation between Risk Score and clinical characteristics. Wilcoxon signed‐rank sum test showed that tumor stage, N stage, and M stage were closely correlated with the Risk Score (*p* < 0.05). While there is a higher Risk Score, there is a higher tumor stage, N stage, and M stage. Age and gender behaved the no correlation with the Risk Score (Figure 3A–F). A heatmap about the relative of two groups and clinical characteristics was also showed in Figure 3G.

### 3.5 A prognostic nomogram can better predict the prognosis of 46 patients

**FIGURE 4:**
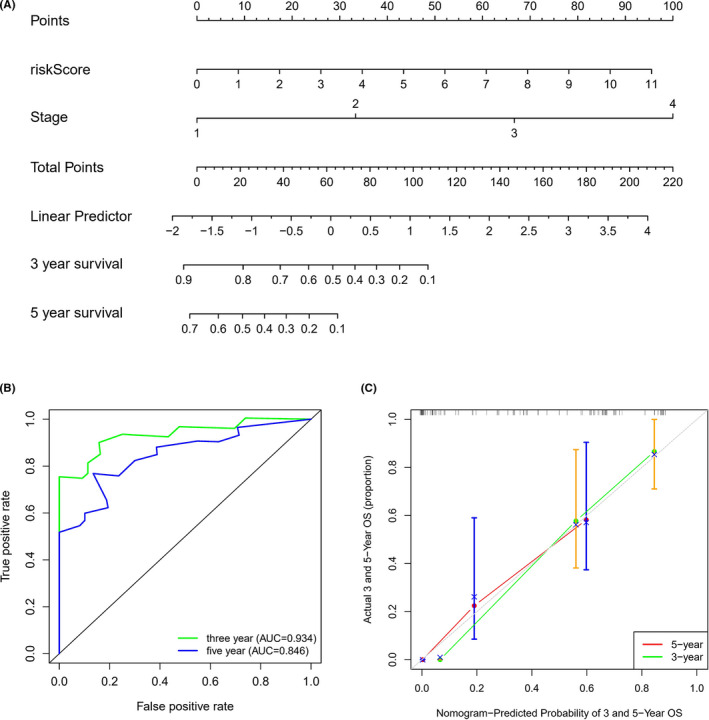
Construction of nomogram for predicting prognosis. (A) Nomogram based on Risk Score and tumor stage. (B) Time‐dependent ROC curves of 3 and 5 years in nomogram. (C) Calibration curve of nomogram

In order to improve the predictive performance, we established a prognostic nomogram, combined the immune Risk Score and stage (Figure 4A). The prognostic nomogram showed better predictive performance than original model of signature with 0.934 of 3‐year‐AUC and 0.846 of 5‐year‐AUC (Figure 4B). Further calibration curve showed that the overall survival proportion in nomogram was consistent with the real overall survival proportion, which proved that the prognostic nomogram could better predict the prognosis of LUAD patients (Figure 4C).

### 3.6 Tumor immune microenvironment of 46 patients

**FIGURE 5:**
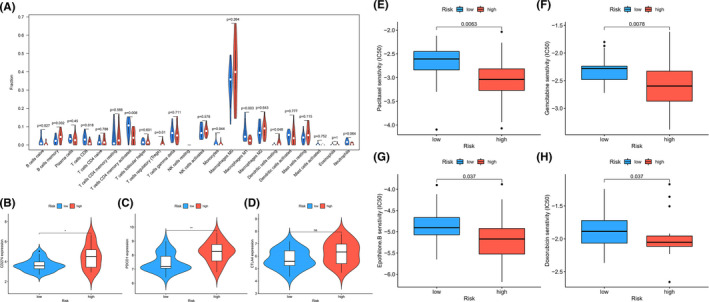
Different immune characteristics of “driver gene negative” LUAD in low‐risk and high‐risk group. (A) Twenty immune cells of microenvironment in low‐risk and high‐risk group. (B–D) Correlations between PD‐1/PD‐L1/CTLA4 and low‐risk and high‐risk group. (E–H) Drug sensitivity analysis in patients of “driver gene negative” LUAD in low‐risk and high‐risk group. *p < 0.05, **p < 0.01, and ***p < 0.001

Since the samples we studied were “driver gene‐negative” LUAD patients and could not benefit from targeted therapy, the characteristics of the immune microenvironment in LUAD patients in high‐risk and low‐risk groups were analyzed in order to obtain immunotherapy's support. Based on CIBESORT deconvolution algorithm, the evaluation results showed that seven immune cells were associated with group difference. In the low‐risk group, B cell naïve, T cells CD4 memory activated, T cell CD8, and Macrophages M1 were highly expressed, while in the high‐risk group, B cell memory, Dendritic cells, and T cells regulatory (Tregs) resting were highly expressed (Figure 5A).

In addition, the expression of immune check point gene (PD‐1/ PD‐L1/CTLA‐4) in 46 LUAD patients were also compared, and we discovered that PD‐1/PD‐L1/CTLA‐4 were higher expressed in the high‐risk group, which might suggest the different immune characteristics and different effect of immunotherapy in the two groups (Figure 5B–D).

### 3.7 Drug sensitivity analysis in patients with “driver gene‐negative” LUAD

According to the Cancer Genome Project drug prediction database, “pRRophetic” package was used to analyze the prediction of response to common drugs for LUAD patients in the high‐risk and low‐risk group. We discovered that doxorubicin, epothilone.B, gemcitabine, and paclitaxel showed statistically significant differences in two groups (*p* < 0.05), and in high‐risk group, the half inhibitory centration (IC50) of doxorubicin, epothilone.B, gemcitabine, and paclitaxel was lower than low‐risk group, which might suggest high‐risk group was more suitable for chemotherapy **(**Figure 5E–H).

## 4 DISCUSSION

The effect of targeted drug therapy for patients with “driver gene positive” lung adenocarcinoma is significant, greatly improving the survival rate of patients with lung adenocarcinoma. Previous clinical trials have demonstrated that, represented by EGFR mutations, TKIs such as erlotinib, gefitinib treatment is encouraging compared with chemotherapy in improving FPS.^15^, ^16^ However, in clinical practice, about 25% of patients with lung adenocarcinoma are “driver gene negative”^17^, ^18^, ^19^, ^20^, ^21^ and cannot benefit from targeted therapy. Although the application of immune checkpoint drugs brings hope to these patients, there are still many problems in clinical application, so new therapeutic targets are especially important for patients with “driver gene negative” LUAD.

Numerous studies have reported that immune‐related genes (IRGs) are tightly associated with the development of various types of malignant tumors and affect the prognosis of cancer patients.^22^, ^23^, ^24^, ^25^ Similarly, IRGs have also been shown to be closely correlated with prognosis of lung adenocarcinoma.^26^, ^27^ Differently, looking at the point of “driver gene negative,” we sought to explore the internal relationship between IRGs and prognosis in patients with this subtype LUAD, and to further analyze the different immune characteristics of this subtype patients in order to seek support for immunotherapy.

In this study, 59 DEIRGs were screened from patients with “driver gene negative” LUAD, and screened 1175 IRGPs with circulating pairing and 0–1 matrix. A marker composed of 5‐IRGPs was obtained by Cox regression analysis. We found CD1A|CXCL13, CD1A|S100A7L2, IFNA7|CMTM2, IFNA7|CSF3, CAMP|TFR2, this 5‐IRGPs were strongly associated with the prognosis of patients, all of which were protective factors for the prognosis of lung adenocarcinoma. Including eight subtypes of genes, the signature suggested that the higher expression of CD1A, IFNA7 and CAMP in CD1A|CXCL13, CD1A|S100A7L2, IFNA7|CMTM2, IFNA7|CSF3 and CAMP|TFR2 led to the higher scores of 5‐IRGPs (5 IRGPs all = 1), the lower Risk Score and the better prognosis. And in clinical work, the detection of expressions of the 5‐IRGPs would be economic and practical instead of examining all RNA's‐specific expression. Then, corresponding treatment would be correctly taken by doctors according to the Risk Score calculated by the 5‐IRGPs. Previous studies have found that multiple genes in these gene pairs play an important role in the development of lung cancer. Some studies have demonstrated that CD1A has antitumor effect and decreased CD1A is associated with early recurrence of lung adenocarcinoma.^28^ A large number of studies have demonstrated that CXCL13 promotes progression of lung cancer and act as a risk factor for lung cancer,^29^, ^30^, ^31^ related studies have also indicated that CSF3 promotes the development of NSCLC by promoting angiogenesis and immunosuppression,^32^ while S100A7L2 (S100a7b), IFNA7, CMTM2, CAMP, and TFR2 are demonstrated that they play an important role in other cancers occurrence and development by regulating the downstream pathway, which provide meaningful reference value for NSCLC.^33^, ^34^, ^35^, ^36^, ^37^, ^38^, ^39^ The model established based on five immune‐related gene pairs could accurately predict the prognosis of patients with negative gene drive, with AUC of 0.850 and 0.810 at 3‐years and 5‐years. Additionally, the AUC of nomogram constructed in combination with tumor stage at 3 and 5 years could reach 0.934 and 0.846. Although there have been previous studies on prediction models of immune‐related genes in lung adenocarcinoma, there are no studies on immune‐related genes for important subtypes of “driver gene‐negative” LUAD. At the same time, unlike previous studies investigating the relationship between expression and prognosis, our use of cyclic pairing and 0–1 matrix can effectively avoid the impact of expression measurement errors, and it may be more persuasive to focus on the expression of genes or not.

Similarly, we explored the correlation between the immune marker and clinical features. The prediction performance of the model based on the marker possess high predictive power. In addition, we found that the Risk Score was correlated with tumor stage, M stage, and N stage, and tumor size was not significantly correlated with the score. The model immune‐related genes we obtained may promote tumor progression at the molecular level.

To further explore the mechanism of predict model, we investigated the immune characteristics of the tumor microenvironment and PD‐1, PD‐L1, and CTLA‐4 expression in patients with lung adenocarcinoma. We found that low‐risk multiple immune cells were highly expressed. High‐risk PD‐1, PD‐L1, and CTLA‐4 expression was higher in the low‐risk group, suggesting that patients in our high‐risk group will be more sensitive to immunotherapy. Finally, we initially performed drug susceptibility analysis of “driver gene negative” lung adenocarcinoma using the Cancer Genome Project drug prediction database, and the results of the analysis suggested that the high‐risk group was more sensitive to doxorubicin, epothilone. B, gemcitabine and paclitaxel. A great concern is that the high‐risk group may be better candidates for immunotherapy, while the low‐risk group has higher sensitivity to chemotherapeutic drugs. This makes it possible to use this model to guide the choice of treatment modalities. We look forward to adding clinical trials in subsequent studies to confirm our study.

The prognosis and treatment of patients with “driver gene negative” LUAD is a difficult point in clinical work. Our exploration is based on single‐center data, and the sample size still needs to be improved. Further biological verification is necessary. At the same time, over the past decade from 2003 to 2015, the treatment guidelines for lung cancer have also changed, affecting the prognosis of some patients, which cannot be avoided in this retrospective study. And it is remarkable that the latest guideline about lung adenocarcinoma suggests patients with “driver gene negative” LUAD receiving immunotherapy,^40^ which might lead to a better prognosis for these patients. There are still no relevant studies in “driver gene negative” lung adenocarcinoma immunotherapy, and we are eager to investigate this in the next clinical work.

## 5 CONCLUSIONS

We propose an intrinsic link between prognosis and IRGs in patients with “driver gene negative” LUAD, construct a prognostic prediction model using 5‐IRGPs, and analyze tumor immune cell infiltration. In addition, the signature can predict patient sensitivity to some chemotherapeutic agents as well as expression of immune checkpoint genes (PD‐1/PD‐L1/CTLA‐4). At the same time, aiming at improving the accuracy of the prediction model and facilitating its clinical application, we combine the immune characteristics and clinical information to construct the nomogram, and achieve good prediction effect.

## CONFLICT OF INTEREST

The authors report no conflicting or competing interests.

## AUTHOR CONTRIBUTIONS

He‐yuan Cai: Data curation, Methodology, Investigation, Writing‐original draft, Writing‐review & editing. Hao‐shuai Yang: Data curation, Methodology, Investigation, Writing‐original draft, Writing‐review & editing. Shi‐chao Shan: Data curation, Investigation, Resources, Writing‐review & editing. Yi‐yan Lei: Data curation, Investigation, Resources, Writing‐review & editing. Jian‐yong Zou: Data curation, Investigation, Resources, Writing‐review & editing. Ying Zhu: Conceptualization, Methodology, Resources, Formal analysis, Writing‐original draft, Writing‐review & editing, Supervision, Funding. Hong‐he Luo: Conceptualization, Writing‐review & editing, Supervision.

## ETHICS STATEMENT

The study involving human participants was reviewed and approved by the Ethics Committee of the First Affiliated Hospital of Sun Yat‐sen University.

## Data Availability

The data are available from the corresponding author upon reasonable request.
